# Machine learning and quantitative computed tomography radiomics prediction of postoperative functional recovery in paraplegic dogs

**DOI:** 10.1111/vsu.70016

**Published:** 2025-10-02

**Authors:** Daniel Low, Scott Rutherford

**Affiliations:** ^1^ Frank. Pet Surgeons Leeds UK; ^2^ Swift Referrals Wetherby UK

## Abstract

**Objective:**

To develop a computed tomography (CT)‐radiomics‐based machine‐learning algorithm for prediction of functional recovery in paraplegic dogs with acute intervertebral disc extrusion (IVDE).

**Study design:**

Multivariable prediction model development.

**Sample population:**

Paraplegic dogs with acute IVDE: 128 deep‐pain positive and 86 deep‐pain negative (DPN).

**Methods:**

Radiomics features from noncontrast CT were combined with deep‐pain perception in an extreme gradient algorithm using an 80:20 train–test split. Model performance was assessed on the independent test set (Test_full_) and on the test set of DPN dogs (Test_DPN_). Deep‐pain perception alone served as the control.

**Results:**

Recovery of ambulation was recorded in 165/214 dogs (77.1%) after decompressive surgery. The model had an area under the receiver operating characteristic curve (AUC) of .9118 (95% CI: .8366–.9872), accuracy of 86.1% (95% CI: 74.4%–95.4%), sensitivity of 82.4% (95% CI: 68.6%–93.9%), and specificity of 100.0% (95% CI: 100.0%–100.0%) on Test_full_, and an AUC of .7692 (95% CI: .6250–.9000), accuracy of 72.7% (95% CI: 50.0%–90.9%), sensitivity of 53.8% (95% CI: 25.0%–80.0%), and specificity of 100.0% (95% CI: 100.0%–100.0%) on Test_DPN_. Deep‐pain perception had an AUC of .8088 (95% CI: .7273–.8871), accuracy of 69.8% (95% CI: 55.8%–83.7%), sensitivity of 61.8% (95% CI: 45.5%–77.4%), and specificity of 100.0% (95% CI: 100.0%–100.0%), which was different from that of the model (*p* = .02).

**Conclusion:**

Noncontrast CT‐based radiomics provided prognostic information in dogs with severe spinal cord injury secondary to acute intervertebral disc extrusion. The model outperformed deep‐pain perception alone in identifying dogs that recovered ambulation following decompressive surgery.

**Clinical significance:**

Radiomics features from noncontrast CT, when integrated into a multimodal machine‐learning algorithm, may be useful as an assistive tool for surgical decision making.

**Abbreviations**
AUC: area under the receiver operating characteristic curve
CT: computed tomography
DICOM: digital imaging and communications in medicine
DPN: deep‐pain negative
GLCM: grey level co‐occurrence matrix
GLRLM: grey level run length matrix
GLSZM: grey level size zone matrix
GLSZM‐LALGLE: gray level size zone matrix‐large area low gray level emphasis
IBSI: image biomarker standardization initiative
IQR: interquartile range
IVDE: intervertebral disc extrusion
MRI: magnetic resonance imaging
NPV: negative predictive value
PACS: picture archiving and communication system
PPV: positive predictive value
SCI: spinal cord injury
Test_DPN_
: test set of deep‐pain negative dogs
Test_full_
: independent test set
TRIPOD: transparent reporting of a multivariable prediction model
XGBoost: eXtreme Gradient Boosting

## 1 INTRODUCTION

Acute thoracolumbar intervertebral disc extrusion (IVDE) is a leading cause of pelvic limb neurological dysfunction in dogs.^1^, ^2^ Pelvic limb neurological deficits, graded using the modified Frankel scale, correlate with spinal cord injury (SCI) severity and functional outcome, making this assessment a reliable and accessible tool for evaluating the severity of SCI.^3^ The absence of deep‐pain perception is indicative of severe SCI and has repeatedly been shown to be a strong negative predictor of functional recovery in dogs with acute thoracolumbar IVDE.^4^, ^5^, ^6^, ^7^

Magnetic resonance imaging (MRI) biomarkers have been investigated extensively but their clinical utility is limited by their lack of specificity, with recent studies that employed high‐field MRI failing to replicate the findings of earlier studies.^3^, ^8^ Magnetic resonance imaging is further limited by prolonged acquisition times, limited availability,^8^ and a failure to show demonstrable improvements in patient outcomes compared to other imaging modalities.^9^ Serum biomarkers and electrophysiological testing have been investigated as alternative biomarkers but are not widely accessible in clinical practice.^10^, ^11^

Radiomics is the study of quantitative medical image analysis aimed at extracting imaging biomarkers for disease diagnosis or prognostication.^12^ Unlike traditional radiology, which relies on qualitative and semantic image interpretation,^13^ radiomics analyses pixel‐level image data to extract quantitative features that are imperceptible to the human eye.^14^ By providing objective and reproducible assessments of tissue characteristics, radiomics is potentially applicable as a noninvasive biomarker across various veterinary disciplines, with prior reports of radiomics as a diagnostic aid in feline enteropathies,^15^ equine musculoskeletal injuries,^16^ and canine brain lesions.^17^, ^18^ However, radiomics generates high‐dimensional data with a large number of extracted features, necessitating the use of machine‐learning techniques for feature selection and predictive modeling.^14^ In veterinary neurosurgery, machine‐learning models trained on structured electronic health‐care data have been shown to achieve moderate to high accuracy in predicting deep‐pain negative (DPN) dogs following acute IVDE.^19^ In the context of dogs with severe SCI, semiquantitative nonradiomics‐based MRI analysis has been investigated previously and was not found to be an accurate prognostic tool.^4^ The only prior study of machine learning in veterinary neurosurgery^19^ incorporated semiquantitative MRI assessment of T2‐weighted spinal‐cord hyperintensity into their predictive model but radiomics otherwise remains uninvestigated in veterinary neurosurgery.

Decompressive surgery is not benign and is associated with a reasonable risk of perioperative morbidity.^20^ The failure to regain ambulation following surgery also has profound consequences for both dogs and owners, often necessitating lifelong management of immobility and double incontinence.^21^ Accurate prognosis is therefore important, particularly in paraplegic dogs and especially in those lacking deep‐pain perception, before committing to invasive and costly decompressive surgery. Apart from MRI, computed tomography (CT) is the alternative cross‐sectional imaging modality for acute IVDE and has the advantage of being more widely available.^2^, ^22^ This study aimed to develop and internally validate machine learning based on quantitative CT radiomics features, and to assess its ability to predict postoperative functional recovery in paraplegic dogs with acute IVDE. The study specifically compared the predictive value of CT‐based radiomics features with deep‐pain perception as a well established prognostic marker.^3^, ^4^ The null hypothesis tested was that CT radiomics features within a machine‐learning algorithm would not differ from the assessment of deep‐pain perception as a prognostic marker.

## 2 MATERIAL AND METHODS

### 2.1 Study design, image acquisition, perioperative care

This study is reported in accordance with the Transparent Reporting of a Multivariable Prediction Model (TRIPOD) guidelines (Supporting Information, File S1). All data were obtained retrospectively with written client consent for the anonymized use of patient information for research purposes.

A retrospective cohort study was conducted via a medical record search of the 2018–2025 neurosurgical caseload of a single veterinary referral hospital in the United Kingdom.

Inclusion criteria were: (1) paraplegic dogs; (2) a diagnosis of acute thoracolumbar IVDE, defined as clinical signs of 10 days or less; (3) availability of Digital Imaging and Communications in Medicine (DICOM) images from noncontrast CT; (4) decompressive surgery performed; (5) a minimum follow up of 28 days. Dogs were excluded if they had undergone prior spinal surgery.

The clinical data that were collected included preoperative neurological grade, age at time of surgery, breed, body weight, sex and neuter status, duration of clinical signs, use of contrast myelography, and attending surgeon. Follow‐up data included time and method of follow up, with binary recovery of ambulation as the primary outcome measure. Dogs were grouped into cohorts based on recovery of ambulation at the last follow up.

Dogs were positioned in dorsal or sternal recumbency with thoracic limbs extended cranially and pelvic limbs extended caudally. The field of view included the vertebral column from the third thoracic vertebrae to the sacrum. All static CT studies of the spine (Revolution ACT 16‐slice; General Electric; Boston, MA, USA) were acquired preoperatively under general anesthesia.

The CT studies were acquired with a tube voltage of 120 kVp, tube current of 40 mA, a 1.2 mm focal spot, a pitch factor of 0.9375, and a slice thickness of 1.25 mm. The sequences were reconstructed with a rescale intercept of −1024, a rescale slope of 1, and standard bone (window center: 500 HU, width: 2000 HU) and soft tissue (window center: 40 HU, width: 400 HU) windows. When indicated, myelography using 0.2–0.3 mL/kg iohexol (Omnipaque 240 mg iodine/ml; GE Healthcare) was performed in the same anesthetic episode, via cisternal or lumbar puncture. Perioperative cefuroxime (Zinacef; GlaxoSmithKline) 20 mg/kg was administered at the discretion of the attending surgeon and was not continued into the postoperative period. Standard hemilaminectomy or pediculectomy was performed via a dorsal approach to decompress the thoracolumbar spinal cord, with the extent of decompression determined through review of noncontrast CT and CT myelography, where used. Same‐level fenestration, multilevel prophylactic fenestration, or durotomy was not performed routinely.

All surgical procedures were performed by board‐certified surgeons (ECVS or ACVS), residents under direct supervision, or nonboarded clinicians. Patients were hospitalized postoperatively for analgesia and nursing care before discharge with nonsteroidal anti‐inflammatory drugs and gabapentin 10 mg/kg, administered orally three times daily, and instructions for 4 weeks of strict cage rest. Follow‐up neurological examination was requested routinely at 4 weeks postoperatively. If this was not possible in person, it was obtained remotely instead. Follow‐up neurological examinations were repeated at nonstandardized intervals until 6 months postoperatively to maximize detection of ambulation recovery. Follow up was unblinded due to the retrospective nature of data collection.

### 2.2 Image segmentation and feature extraction

**FIGURE 1:**
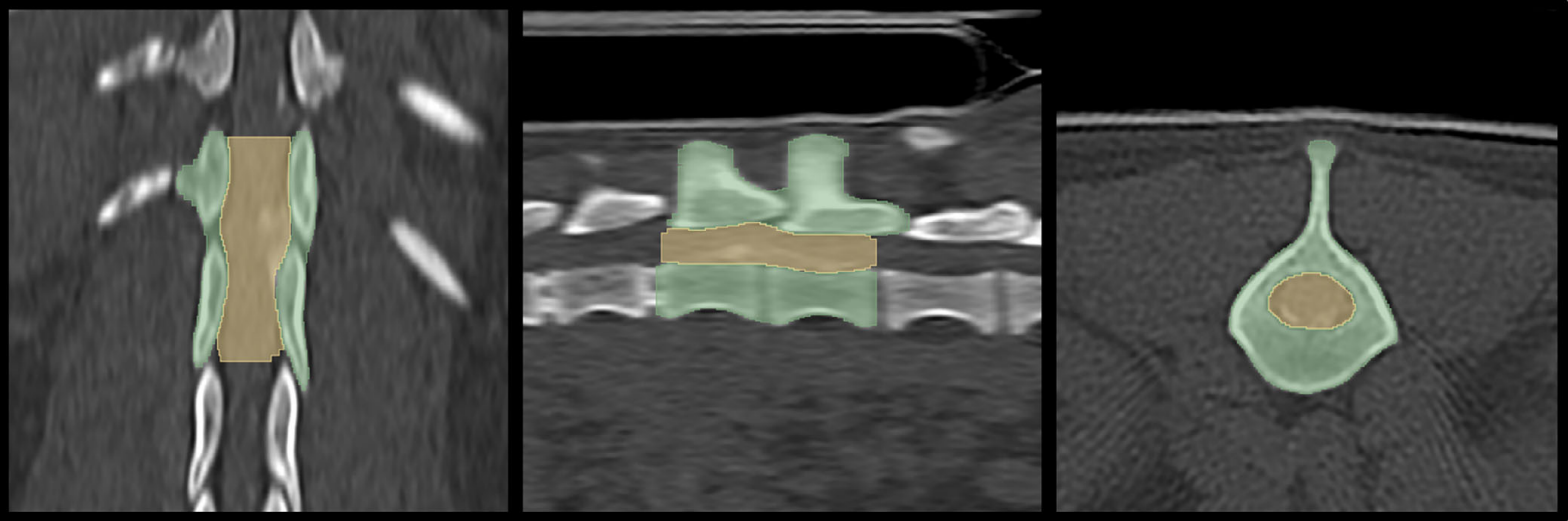
Multiplanar reconstruction (left, dorsal; middle, sagittal; right, transverse) in a bone window showing segmentation of the lesion epicenter in a dog with an acute T13‐L1 intervertebral disc extrusion. The vertebral column is segmented in green and the spinal canal is segmented in yellow.

Noncontrast CT sequences were exported from the hospital picture archiving and communication system (PACS) (Easyvet; VetZ, Isernhagen, Germany) in DICOM format, reconstructed in both bone and soft tissue windows. Image segmentation was performed using open‐source software (Slicer 5.6.2).^23^ The lesion epicenter was defined based on a modification of a previous study^4^ to account for the different cross‐sectional imaging modality. Specifically, the lesion epicenter was identified as the spinal segment requiring surgical decompression, as determined by the attending surgeon, extending from the cranial endplate of the most cranially affected vertebra to the caudal endplate of the most caudally affected vertebra (Figure 1). The vertebral column and spinal canal overlying the lesion epicenter were segmented manually using Hounsfield unit thresholding. Segmentations were jointly smoothed using a 3 mm kernel. The vertebral column was segmented in a bone window reconstruction and the spinal canal segment was segmented in a soft tissue window reconstruction.

Radiomics features were extracted using an Image Biomarker Standardization Initiative (IBSI)‐compliant Python package.^24^ Features were obtained separately for the vertebral column and spinal canal segments without resampling, using a 1.0 mm Laplacian of Gaussian kernel, a bin width of 10.0, and a symmetrical gray level co‐occurrence matrix. A total of 107 quantitative radiomics features were extracted per segment, comprising 14 shape features, 18 first‐order features, and 75 second‐order features (Supporting Information, File S2).

### 2.3 Machine learning and statistical analysis

The 107 features from each segmentation were tabulated and preoperative neurological grade was included as a covariate, resulting in a total of 215 features for statistical analysis and machine learning. Data normality was assessed using the Shapiro–Wilk test. Parametric data are reported as means and standard deviations. Nonparametric data are reported as medians and interquartile ranges (IQR).

Univariable statistical analysis was used to compare individual radiomics features between cohorts, with the Benjamini–Hochberg correction applied to control for multiple testing. Dataset balancing was performed with the synthetic minority oversampling technique with a 1:2 minority‐to‐majority class ratio prior to machine learning. The dataset was randomly split into training and testing sets in an 80:20 ratio using a pseudorandom split function with a fixed seed.

The model was based on eXtreme Gradient Boosting (XGBoost). It was trained and tuned witfh automated Bayesian hyperparameter tuning, using the area under the receiver operating characteristic curve (AUC) as the target maximizing metric. The model was internally validated on the independent test set with all paraplegic dogs (Test_full_), and then with deep‐pain negative dogs only (Test_DPN_). Predictive performance was reported as AUC, accuracy, sensitivity, specificity, positive predictive value (PPV), and negative predictive value (NPV). Sensitivity and specificity were defined as the algorithm's ability to predict recovery and nonrecovery of ambulation respectively. Fisher's exact test was used to calculate these metrics for deep‐pain perception as a binary predictor of postoperative outcome. Nonparametric bootstrapping with 1000 resampling iterations estimated 95% confidence intervals for each metric. Model performance was compared with deep‐pain perception using McNemar's test.

Feature frequency scores (F‐score) were calculated within XGBoost to identify features contributing the most to algorithm performance. F‐scores represent the number of times a feature is used to split the data across all trees in the model, serving as a proxy for its relative contribution to decision making. Higher F‐scores indicate frequent use in informative splits, but do not indicate the direction or consistency of the association with the outcome. Statistical significance was defined as *p* < .05.

All statistical analysis and data visualization were performed with *pandas 2.2.2, numpy 1.26.4*, *scipy* 1.13.1, *seaborn* 0.13.2, *matplotlib 3.10.0*, *statsmodels* 0.14.4, *sklearn* 1.6.1, *imblearn* 0.13.0, *xgboost* 2.1.4, and *optuna* 4.2.1 in Python version 3.11.11.^25^, ^26^, ^27^, ^28^, ^29^, ^30^, ^31^, ^32^, ^33^, ^34^, ^35^

## 3 RESULTS

The medical record search identified 234 paraplegic dogs with acute thoracolumbar intervertebral disc extrusion that underwent decompressive surgery; all eligible cases during the study period were included. Seven dogs were excluded for previous spinal surgery, three dogs for corrupted DICOM archives, and 20 for a lack of follow up (12 died or were euthanized and no follow‐up data were available for eight), resulting in a sample population of 214 dogs. Dachshunds (*n* = 74) and French bulldogs (*n* = 65) were the most common breeds in the dataset. There were 122 males (60 neutered) and 92 females (44 neutered). Median age was 4.5 years (IQR: 3.8–5.8 years) and median body weight was 10.6 kg (IQR: 7.6–14.2 kg). Median duration of onset of clinical signs was 2 days (IQR: 1–3 days).

Myelography was performed in 65/214 dogs (30.4%). The attending surgeon was board certified in 127/214 cases (59.3%), a resident under direct supervision in 24/214 (11.2%), and a nonboarded clinician in 63/214 (29.4%).

There were 128 dogs (59.8%) with deep‐pain perception and the remaining 86 dogs (40.1%) were DPN. Follow up was obtained via neurological examination in 197/214 cases (92.1%) and via telephone in the remaining 17/214 (7.9%) with a median follow‐up time of 37 days (IQR: 30–54.5 days). The rate of recovery of ambulation after decompressive surgery in this sample population was 165/214 dogs (77.1%). The deep‐pain positive subgroup had a recovery rate of 123/128 dogs (96.1%) and the DPN subgroup had a recovery rate of 42/86 (48.8%).

None of the 214 radiomics features was associated with recovery of ambulation on univariable analysis (File S3). Final model parameters after Bayesian hyperparameter optimization are provided in Supporting Information, File S4. On Test_full_, the model achieved an AUC of 0.9118 (95% CI: 0.8366–0.9872), an accuracy of 86.1% (95% CI: 74.4% –95.4%), a sensitivity of 82.4% (95% CI: 68.6%–93.9%), a specificity of 100.0% (95% CI: 100.0%–100.0%), a PPV of 100.0% (95% CI: 100.0%–100.0%), and an NPV of 60.0% (95% CI: 33.3%–83.3%). On Test_DPN_, the model demonstrated an AUC of 0.7692 (95% CI: 0.6250–0.9000), an accuracy of 72.7% (95% CI: 50.0%–90.9%), a sensitivity of 53.8% (95% CI: 25.0%–80.0%), a specificity of 100% (95% CI: 100.0%–100.0%), a PPV of 100% (95% CI: 100.0%–100.0%), and an NPV of 60.0% (95% CI: 33.3%–85.7%). On Test_full_, DPP showed an AUC of 0.8088 (95% CI: 0.7273–0.8871), an accuracy of 69.8% (95% CI: 55.8%–83.7%), a sensitivity of 61.8% (95% CI: 45.5%–77.4%), a specificity of 100.0% (95% CI: 100.0%–100.0%), a PPV of 100.0% (95% CI: 100.0%–100.0%), and an NPV of 40.9% (95% CI: 22.7%–63.2%). The model outperformed DPP in predicting recovery of ambulation (*p* = .02; Table 1).

**TABLE 1:** Model performance (95% CI) on Test_DPN_ and Test_full_, compared with deep‐pain perception.

Performance metric	Predictive model		Deep‐pain perception
	Test_DPN_	Test_full_	Test_full_
AUC	.7692 (.6250–.9000)	.9118 (.8366–.9872)	.8088 (.7273–.8871)
Accuracy	72.7% (50.0%–90.9%)	86.1% (74.4% –95.4%)	69.8% (55.8%–83.7%)
Sensitivity	53.8% (25.0%–80.0%)	82.4% (68.6%–93.9%)	61.8% (45.5%–77.4%)
Specificity	100.0% (100.0%–100.0%)	100.0% (100.0%–100.0%)	100.0% (100.0%–100.0%)
PPV	100.0% (100.0%–100.0%)	100.0% (100.0%–100.0%)	100.0% (100.0%–100.0%)
NPV	60.0% (33.3%–85.7%)	60.0% (33.3%–83.3%)	40.9% (22.7%–63.2%)
McNemar's test		***p* = .02**	

Abbreviations: AUC, area under the receiver operating characteristic curve; NPV, negative predictive value; PPV, positive predictive value, TestDPN; independent test set, Testfull, test set of deep‐pain negative dogs.

**FIGURE 2:**
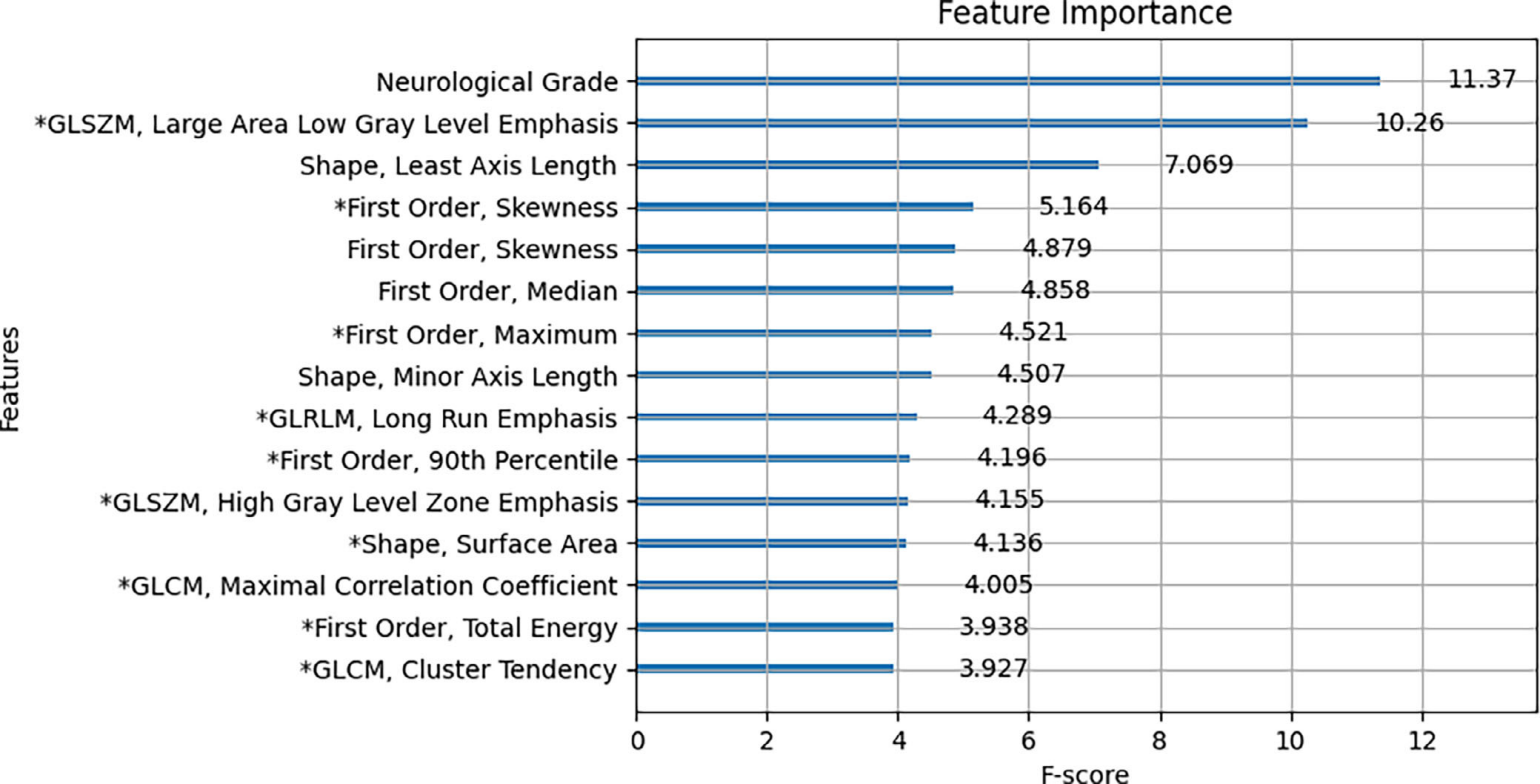
Feature importance plot illustrating the F‐scores of the 15 features contributing most to algorithm predictive performance (* indicates features obtained from the spinal canal). GLSZM, grey level size zone matrix; GLRLM, grey level run length matrix; GLCM, grey level co‐occurrence matrix.

Neurological grade was the single most important feature in the model's decision‐making process, with an F‐score of 11.37 (Figure 2). The cumulative F‐scores for spinal canal features and vertebral column features were 48.59 and 21.31 respectively. Unrepresented features were considered to be minimally important. Summary statistics of the 15 most important features, except for neurological grade, are reported in Table 2.

**TABLE 2:** Summary statistics of the features with the highest F‐scores between cohorts, represented as medians (IQRs)

		Recovered	Did not recover
Spinal canal features	GLSZM—Large Area Low gray level emphasis	47.08 (22.23–92.08)	77.48 (21.79–113.1)
	First order—skewness	1.740 (1.433–2.013)	1.769 (1.628–1.996)
	First order—maximum	663.0 (614.0–732.0)	688.0 (607.0–784.0)
	GLRLM—long run emphasis	2.316 (2.116–2.546)	2.360 (2.076–2.618)
	First order—90th Percentile	233.0 (209.0–263.0)	229.0 (201.0–265.0)
	GLSZM—high gray level zone emphasis	961.5 (873.0–1114)	970.6 (834.4–1207)
	Shape—surface area	1227 (939.5–1729)	1623 (1174–2025)
	GLCM—maximal correlation coefficient	0.8709 (0.8382–0.8905)	0.8685 (0.8449–0.8886)
	First order—total energy	39 384 497 (28654306–54 115 024)	41 888 367 (33 388 856–59 497 543)
	GLCM—cluster tendency	235.0 (193.5–314.3)	220.8 (173.8–302.0)
Vertebral column features	Shape—least axis length	20.68 (18.49–23.61)	21.60 (18.27–24.96)
	First order—skewness	0.8463 (0.7063–0.9745)	0.7964 (0.6719–0.9623)
	First order—median	643.0 (586.0–690.0)	658.0 (595.0–682.0)
	Shape—minor axis length	26.59 (23.35–31.63)	29.55 (23.96–35.01)

Abbreviations: GLSZM, grey level size zone matrix; GLRLM, grey level run length matrix; GLCM, grey level co‐occurrence matrix.

## 4 DISCUSSION

This study demonstrates that quantitative CT radiomics features within a machine‐learning algorithm, coupled with neurological grade, may offer prognostic information in paraplegic dogs after surgical decompression for acute thoracolumbar IVDE. It showed that the model's performance was superior to that of deep‐pain perception as a prognostic marker and therefore, the null hypothesis was rejected.

Machine learning without radiomics has been investigated in a similar patient population with structured health‐care data.^19^ Radiomics analysis integrated with machine learning has been investigated in other areas of veterinary medicine^15^, ^16^, ^17^, ^18^ but not in the context of neurosurgical outcome prediction in dogs. This study therefore represents the first evaluation of radiomics and machine learning in dogs with severe SCI secondary to acute IVDE. Radiomics analysis of CT imaging has been investigated in human neurosurgery and has been shown to be diagnostically accurate and prognostically useful.^36^, ^37^

In dogs with acute thoracolumbar IVDE, the loss of deep‐pain perception has been repeatedly shown to be the most reliable prognostic indicator, with conflicting evidence regarding the utility of other clinical, biochemical, and imaging markers.^3^ A previous study compared semiquantitative nonradiomics MRI analysis to DPP in paraplegic dogs, and showed that DPP was superior to the imaging biomarkers investigated.^4^ In general, MRI has not been proven to be an accurate prognostic marker in dogs with severe SCI, with regards to recovery of ambulation.^3^ Furthermore, radiomics analysis is performed after acquisition and does not require specialized hardware or software, unlike certain nonroutine MRI sequences. This study demonstrates that CT radiomics provides useful data that can be integrated meaningfully in a machine‐learning pipeline to improve prognostication in a population of paraplegic dogs, and provides evidence that non‐MRI imaging modalities are appropriate and useful for imaging acute thoracolumbar IVDE.^9^

Machine‐learning algorithms identify patterns in tabular data such as electronic health records^19^ or quantitative radiomics features and their association with a target outcome. The nature of machine learning allows complex real‐world interactions to be modeled, which may not be evident from traditional statistical analysis.^38^

This study shows that none of the CT radiomics features were associated with postoperative functional outcome on univariable analysis. However, when incorporated into a tree‐based model, the cumulative effect of multiple weak predictors resulted in a strong prediction model.

An XGBoost model was used as it has been shown to excel as a general purpose classifier, and outperforms other tree‐based models in nonmedical tasks^32^ and in veterinary neurosurgery specifically.^19^ The relatively high feature‐to‐sample ratio presented a risk of overfitting, though this was mitigated through the use of XGBoost's inbuilt regularization and feature selection capabilities. Internal validation was performed and demonstrated model generalizability to an extent, but external validation on a fully independent cohort is required to confirm this. The radiomics features collected were aligned to the IBSI standards and extracted with open‐source software, in order to ensure reproducibility in future studies. The CT protocols were reported transparently and, even if alternative hardware or scanning protocols are used, batch‐harmonization techniques exist to account for systematic differences between institutional facilities and protocols,^39^ and controlling for the confounding introduced by external multicenter validation remains feasible.

The Gray Level Size Zone Matrix—Large Area Low Gray Level Emphasis (GLSZM‐LALGLE) feature extracted from the spinal canal played a substantial role in the model. The GLSZM‐LALGLE is a quantitative measure of the distribution of low gray zones within an image segment.^40^ Higher GLSZM‐LALGLE values have been associated with increased histopathologic tissue infiltrates in bone marrow disease.^41^ Although this feature has not been studied specifically in canine myelopathies, a higher GLSZM‐LALGLE value may indicate that a segment has larger regions of hypoattenuating tissue in the context of CT. The results of this study show a trend towards higher GLSZM‐LALGLE values in the cohort of dogs that failed to recover ambulation, and that this may be a potential CT biomarker of spinal cord edema, inflammation, or necrosis.

Shape features from both the spinal canal and vertebral column generally trended towards higher median values in the nonrecovered cohort, compared to the recovered cohort. Shape features are geometric measures of the segmented region^42^ and this may suggest that compressive lesions which were larger or more irregular may be associated with a poorer outcome. First‐order features from the spinal canal and vertebral column represent the characteristics of voxel intensities through the segmented anatomical region, and second‐order features quantify spatial relationships of voxels within the segmented tissues.^42^ Both these groups of features are measures of tissue heterogeneity.

There is limited direct investigation of CT‐radiomics and spinal cord pathology in both humans and dogs but there is evidence that these radiomics features identify tissue heterogeneity within neoplastic tissue outside the central nervous system,^43^, ^44^ and that this analysis may be detecting tissue textural differences that are not grossly visible. The first‐ and second‐order features that the model deemed important did not differ on gross inspection of summary statistics and on statistical analysis; however, the model was able to utilize these textural features towards predicting postoperative outcome.

Feature importance can be visualized but a key limitation of machine learning is interpretability. The influence of a feature on the outcome may vary depending on the context, as machine‐learning models capture complex, nonlinear interactions among multiple variables rather than relying on simple, direct associations.

The F‐scores derived from the spinal canal segmentation indicate that this anatomical compartment contributed the most to the model's decision making. This supports the biological plausibility of the model, as pathology within the spinal canal is directly relevant to neurological outcome. However, the segmentation methodology in this study does not allow for precise localization of pathology within the spinal canal, nor does it distinguish between specific tissue types contributing to the radiomics signal.

The spinal canal segment was defined using the vertebral endplates as consistent anatomical landmarks, encompassing both compressed and noncompressed spinal cord, extruded nucleus pulposus, and to a lesser extent, cerebrospinal fluid and epidural fat. Similarly, the vertebral column segmentation primarily consisted of bone but also included intervertebral disc structures, resulting in contributions from both the annulus fibrosus and in situ nucleus pulposus.

More precise tissue segmentation may offer additional information about the precise source of the radiomics signal but would likely require MRI to achieve this level of anatomical resolution. Noncontrast CT was selected in this study as it was available for all dogs meeting inclusion criteria. Although contrast myelography was performed in approximately one‐third of cases, its use was not standardized and was performed on a selected basis. Additionally, myelographic contrast flow may be influenced by the degree of spinal cord compression and other procedural factors unrelated to the lesion itself, limiting its utility in this study. Future prospective studies with systematic CT myelography may provide more detailed radiomics information but the current research question was best served by using noncontrast CT, ensuring consistency across cases.

Radiomics features such as shape and first‐order statistics are inherently influenced by the size of the segmentation, introducing potential bias in traditional univariable statistical analysis. However, this is less relevant in the context of machine learning, as the XGBoost model accounts for lesion size as a covariate during model training. Differences in segmentation size are therefore unlikely to have introduced bias in model predictions. Further work using more precise segmentation techniques may clarify the relative contribution of different tissues to radiomics feature extraction.

Although this is the first report of its kind, the model developed and internally validated in this study may have potential clinical use, showing high specificity and PPV. It was highly accurate in predicting recovery of ambulation across all paraplegic dogs and in the test sets correctly identified all dogs who regained functional ambulation, including all DPN dogs. In a clinical setting, this provides prognostic information previously unavailable to clinicians. Prior studies have primarily focused on identifying negative prognostic factors^45^, ^46^, ^47^ with limited reporting of positive prognostic indicators. This gap is, in part, due to the limitations of traditional statistical methods in medical outcome prediction. A highly specific model with a high PPV essentially identifies paraplegic dogs with positive prognoses and provides novel information for neurosurgical decision making. For the first time, veterinary neurosurgeons may be able to identify dogs with severe SCI who are highly likely to benefit from surgical decompression and thus tailor client recommendations appropriately.

Anecdotally, the uncertainty of postoperative outcome in DPN dogs is a major barrier to treatment and discourages certain clients from consenting to costly decompressive surgery with a substantial out‐of‐hospital care burden. On the other hand, the model was only moderately sensitive when predicting a failure to make a functional recovery, and these metrics were associated with wide confidence intervals. In clinical practice, it is therefore not advisable to overemphasize a negative prediction from the model. Apart from considering raw algorithm performance, the model is not immediately applicable in clinical practice because of the limitations of manual segmentation and feature extraction, which are time consuming and require specific domain knowledge.

The general consensus among veterinary neurosurgeons is that this population of paraplegic dogs benefits from urgent surgical decompression^8^ and it is therefore impractical to integrate the model into a clinical workflow in its current form. The development of semi‐ or fully automated methods of anatomical segmentation and radiomics feature extraction is advanced in human radiology,^48^ and canine‐specific segmentation models will enable the model and similar algorithms to be integrated into the clinical workflow.

The sample population in this study was generally representative of dogs affected by thoracolumbar IVDE, with young to middle‐aged chondrodystrophic dogs overrepresented. The sample size also represents one of the largest studies of paraplegic dogs with IVDE. The rates of recovery of ambulation of deep‐pain positive and DPN dogs in this study was also within previously reported limits.^8^, ^49^ However, this study's retrospective nature introduced bias with the nonsystematic use of myelography, which was performed at the attending surgeon's discretion after review of noncontrast CT images. Noncontrast CT differs from CT‐myelography in its diagnostic sensitivities^50^ and this would have been an uncontrolled bias within this study. Both noncontrast CT and CT‐myelography also differ from MRI^51^ and may have underestimated the full extent of extradural compression and therefore the definition of the lesion epicenter in this study. Surgery was also performed on a nonrandomized basis.

Some paraplegic dogs may have been managed nonsurgically or euthanized, for clinical or other reasons, and this would have biased the sample population in this study. The quality of surgical decompression and use of adjunctive surgical procedures was uncontrolled, and would have been another source of bias.

Postoperative loss to follow up may have introduced uncontrolled bidirectional bias into the results, although this bias is likely minimal with a loss rate of less than 10%. This outcome measure in this study differed from other studies^4^, ^47^ in that other studies assessed 28‐day recovery of ambulation while the current study assessed recovery of ambulation with a minimum 28‐day follow up. This limits direct comparison between studies and may have missed dogs with delayed recovery of ambulation, which can take up to 6 months.^3^ Although most dogs were assessed via neurological examination, a minority of dogs were assessed remotely, which affects the quality and certainty of outcome data.

This study had a large sample size but it remains a relatively limited sample for machine learning. Dividing the data into separate training and test sets reduced the number of cases in the test set, so model performance was assessed on a small sample of paraplegic dogs only. External validation is therefore warranted.

The study developed a prediction model for recovery after severe SCI in dogs using both neurological grade and quantitative CT radiomics features. This represents a preliminary demonstration of multimodal machine learning in veterinary neurosurgery, where multisource algorithmic input enhances modeling of complex real‐world interactions. Deep‐pain perception is a well established prognostic marker and can be readily assessed on neurological examination. It is therefore unrealistic to assess model performance solely on CT radiomics features by withholding deep‐pain perception from the model.

Multimodal machine learning has been investigated in feline enteropathies^15^ and cervical SCI in humans^52^ with good results. Clinical data within a machine learning model have also been shown to accurately predict postoperative outcome in deep‐pain negative dogs with acute IVDE.¹⁹ Combining quantitative radiomics features with clinical data may further improve prognosis in dogs with severe SCI and mitigate the limitations of this study, which did not account for clinical variables. Investigation of MRI radiomics in acute IVDE may also yield additional pathophysiological or prognostic insights. With external validation and the development of automated segmentation and feature extraction tools, these technologies could be integrated into the veterinary neurosurgeon's workflow to enhance surgical decision making.

In conclusion, this study shows that noncontrast CT contains useful information to aid prognosis in dogs with severe SCI secondary to acute IVDE. Specifically, it showed that quantitative CT radiomics features and neurological grade, within the model, accurately identifies dogs with severe SCI that go on to make a functional recovery after decompressive surgery. It shows that the model outperforms deep‐pain perception as a means of prognosis. We discussed the pathophysiological mechanisms that may underpin radiomics feature extraction and algorithm decision making in this patient population, the potential clinical utility of the model, and the further research required before clinical implementation.

## CONFLICT OF INTEREST STATEMENT

The authors declare no conflict of interest.

## Supporting information


**Data S1:** Supporting Information.

## Data Availability

The raw data, raw statistical analyses, and model parameters which support the conclusions of this study are available as supplementary files.
